# Structural dynamics of calmodulin-ryanodine receptor interactions: electron paramagnetic resonance using stereospecific spin labels

**DOI:** 10.1038/s41598-018-29064-8

**Published:** 2018-07-16

**Authors:** Cheng Her, Andrew R. Thompson, Christine B. Karim, David D. Thomas

**Affiliations:** Department of Biochemistry, Molecular Biology, and Biophysics, University of Minnesota, Minneapolis, Minnesota 55455 USA

## Abstract

We have used electron paramagnetic resonance, with rigid and stereospecific spin labels, to resolve structural states in calmodulin (CaM), as affected by binding of Ca and a CaM-binding peptide (RyRp) derived from the ryanodine receptor (RyR), the Ca channel that triggers muscle contraction. CaM mutants containing a pair of cysteines in the N-lobe and/or C-lobe were engineered and labeled with a stereospecifically bound bifunctional spin label (BSL). RyRp was synthesized with and without TOAC (a stereospecifically attached spin-labeled amino acid) substituted for a single amino acid near the N-terminus. Intramolecular DEER distance measurements of doubly-labeled BSL-CaM revealed that CaM exists in dynamic equilibrium among multiple states, consistent with open, closed, and compact structural models. Addition of RyRp shifted the equilibrium partially toward the compact state in the absence of Ca, and completely toward the compact state in the presence of Ca, supporting a conformational selection model. Inter-protein distance measurements show that Ca stabilizes the compact state primarily by inducing ordered binding of the CaM N-lobe to RyRp, while only slightly affecting the C-lobe. The results provide insight into the structural mechanism of CaM-mediated RyR regulation, while demonstrating the power of using two types of rigidly and stereospecifically bound spin labels.

## Introduction

**Figure 1:**
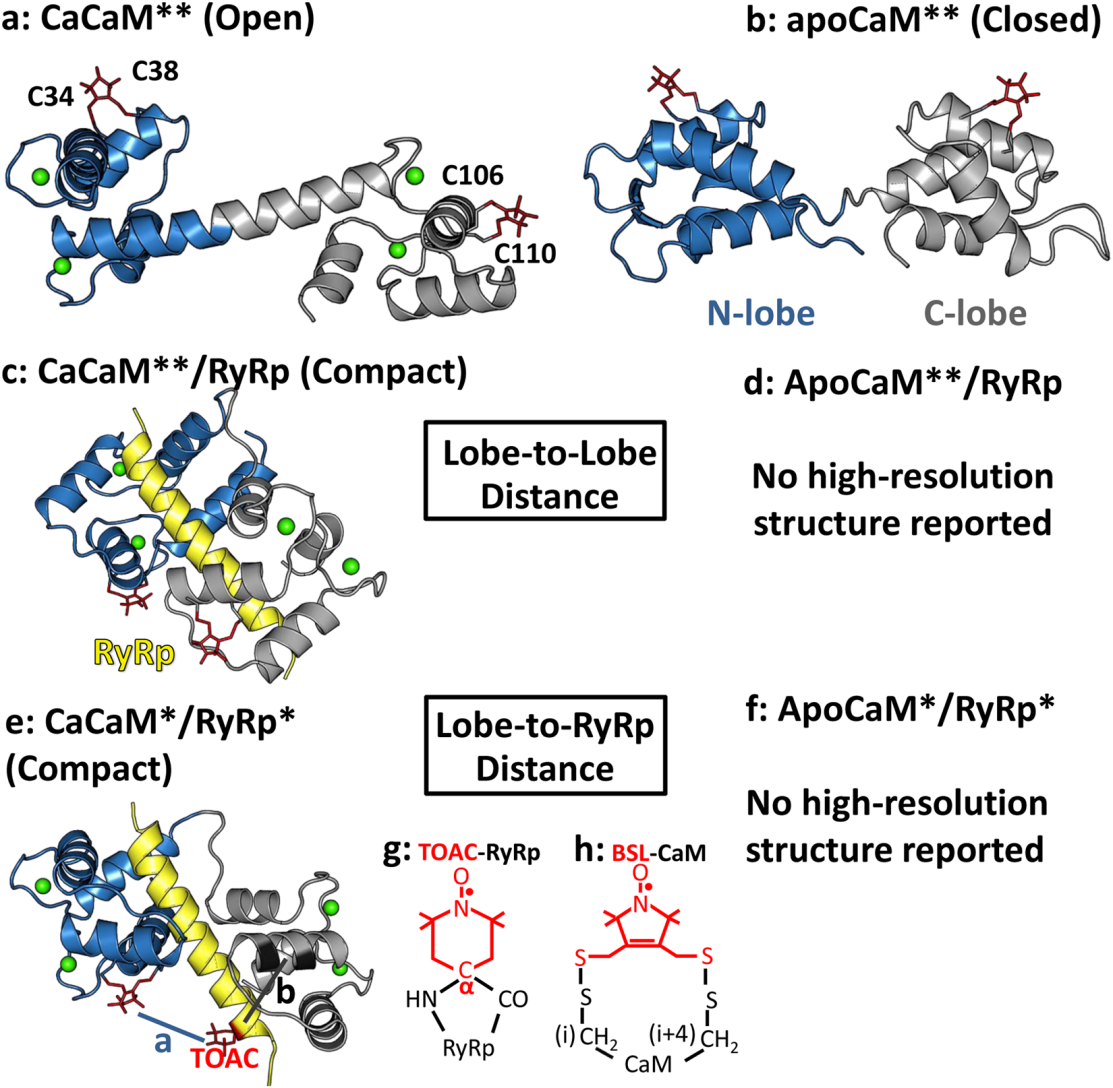
Strategy for spin-spin distance measurements. (**a–d**) CaM lobe-to-lobe intramolecular distances. (**e,f**) CaM lobe-to-RyRp intermolecular distances. Ribbon diagrams produced with PyMOL (www.pymol.org) show CaM N-lobe blue, CaM C-lobe gray, RyRp yellow, spin labels (modeled as in^28^) red. (**a**) CaCaM (PDB: 1CLL)^4^. (**b**) apoCaM (PDB: 1CFD)^8^. (**c,e**) CaCaM/RyRp complex (PDB: 2BCX)^15^. (**d,f**) No high-resolution structures have been reported for apoCaM/RyRp complex. **For a–d, CaM was labeled with BSL (**h**) in the N lobe (T34C, S38C) and in the C-lobe (R106C, T110C). *For e and f, RyRp was labeled with TOAC (**g**) at position 5, and CaM was labeled with BSL (**h**) in the N lobe (distance a) or C lobe (distance b).

Calmodulin (CaM) is a 148-amino acid Ca-binding protein that functions as a regulator of many crucial Ca-dependent cellular and molecular processes, including the release of Ca from sarcoplasmic reticulum (SR) to initiate muscle contraction^1,2^. CaM consists of two canonical EF-hand lobes tethered by a flexible helical linker^3^. The two lobes share high sequence homology (75%) and structural similarity in the presence^4–6^ and absence of Ca^7–9^. However, the differences in the two lobes result in distinct biochemical properties. The C-lobe coordinates Ca ions with 10-fold higher intrinsic affinity^10,11^. The flexible linker between the two lobes enables flexibility that facilitates interaction with a diverse array of targets. One CaM target of growing interest is the tetrameric sarcoplasmic reticulum Ca release channel, the ryanodine receptor (RyR). CaM directly binds to RyR and influences channel opening probability in an isoform-specific manner. In skeletal muscle (RyR1) CaM potentiates channel opening below μM Ca and inhibits above μM Ca; in the cardiac isoform (RyR2) CaM is inhibitory at all Ca levels^12–14^. The goal of the present study is to increase our understanding of the structural changes involved in the Ca-dependent regulation of RyR1. Three high-resolution structural models relating to this problem have been characterized. The X-ray crystal structure of CaCaM revealed an open extended conformation with the N- and C-lobes separated by a folded α-helix central linker region^4^ (Fig. 1a). The solution NMR structure of apoCaM showed a more closed structure, with the central helix partially unfolded^8^ (Fig. 1b). The X-ray crystal structure of CaCaM complexed with a peptide constituting the CaM binding site on RyR (CaCaM/RyRp), showed an even more compact structure, in an anti-parallel configuration^15^ (Fig. 1c).

However, these high-resolution structures leave significant gaps in our understanding of this important system. First, there is no high-resolution structure of the apoCaM/RyRp complex (Fig. 1d and f). Second, it is likely that the structural states in solution are more complex than depicted in Fig. 1, with multiple structural states being occupied at equilibrium, as suggested by NMR data on apoCaM^5,8,9,16,17^.

Structure determination by X-ray crystallography requires trapping a single structural state. While NMR can provide evidence for structural heterogeneity and dynamics, reliable structure determination by NMR typically requires population of a single well-defined structural state. CaM is a flexible signaling protein with a versatile repertoire of structural and functional states, influenced by the binding of Ca and target proteins. The challenge remains to detect and resolve these dynamic structural changes. Distance measurements between spin or fluorescent probes can resolve multiple protein structural states, based on specific techniques in EPR (DEER, double electron-electron resonance, e.g^18^.) or fluorescence (TR-FRET, time-resolved fluorescence resonance energy transfer; e.g^19^.).

We previously used DEER to measure the distribution of interspin distances on CaM spin-labeled at positions T117C (C-lobe) and T34C (N-lobe). That work showed that both in the presence and absence of bound Ca, CaM simultaneously populates both closed (4 nm lobe separation) and open (6 nm lobe separation) structural states, with Ca shifting the equilibrium to the open state^20^. However, it was not possible to compare the structures in that work rigorously with the previously obtained high-resolution structures, because the spin labels used were conventional probes flexibly attached to single Cys residues.

Therefore, in the present study we have employed “stereospecific” spin labels that offer rigid and specific attachment relative to the peptide backbone. For RyRp, we used solid-phase peptide synthesis to incorporate TOAC, a spin-labeled amino acid in which the nitroxide-containing piperidine ring includes the α-carbon (Fig. 1g) and thus directly reports backbone structural dynamics^21–23^. Although TOAC is an unnatural amino acid from the family of Cα,α-disubstituted glycines^24^, it has been shown to have very useful applications for analyzing peptide backbone dynamics^21–23,25^. In recent work, we used EPR of TOAC to detect the Ca-dependent structural dynamics of spin-labeled RyRp bound to CaM^25^. We showed that the structural dynamics of the CaM-bound RyRp is Ca-dependent and non-uniform along the peptide. While compact and immobilized, TOAC requires incorporation via peptide synthesis, which is usually not feasible for molecular weights above 6 kDa.

Therefore, in the present study, to incorporate spin labels stereospecifically into the 16.7 kDa CaM protein, we used mutagenesis to express double-Cys mutants in the N-lobe and C-lobe and labeled each site with a bifunctional spin label (BSL, 3,4-bis-(methanethio-sulfonylmethyl)−2,2,5,5-tetramethyl-2,5-dihydro-1H-pyrrol-1-yloxy) (Fig. 1h). BSL, like TOAC, offers rigid and specific attachment relative to the peptide backbone^26^. When attached to a helix with Cys residues engineered at i and i + 4, BSL attains a well-defined orientation with respect to the helix axis^27^, allowing for precise measurement of helix orientation in an oriented sample^28^. The conformational restriction afforded by the second Cys linker also allows for precise (sub-nanometer accuracy) measurement of distance distributions in dipolar EPR experiments, virtually eliminating contributions from probe flexibility in the measured distance distributions^29^. The present study is the first report in which TOAC and BSL have been used together to measure distances within a protein complex.

The principal technique used in the present study is DEER, which is most sensitive to interspin distances from 2 to 8 nm but has been shown to successfully measure distances up to 16 nm^30^. DEER has sufficient resolution to detect multiple distances simultaneously, quantitating their mean values, disorder, and populations^31,32^. Here we have used DEER to determine (a) the intramolecular lobe-to-lobe distance between the two BSL as a function of Ca and RyRp binding (Fig. 1a–d), and (b) the intermolecular distance between singly labeled N-lobe or C-lobe BSL-CaM and TOAC-labeled RyRp (Fig. 1e,f). This study also employed continuous wave (CW) dipolar EPR, which is sensitive to distances between 0.8 and 2.0 nm^33^, to provide additional information on shorter distances. The results provide new structural insight into the Ca-dependent interactions of CaM and RyR.

## Results

### Distance prediction and data simulation from CaM structural models reveal ideal BSL sites for DEER

**Figure 2:**
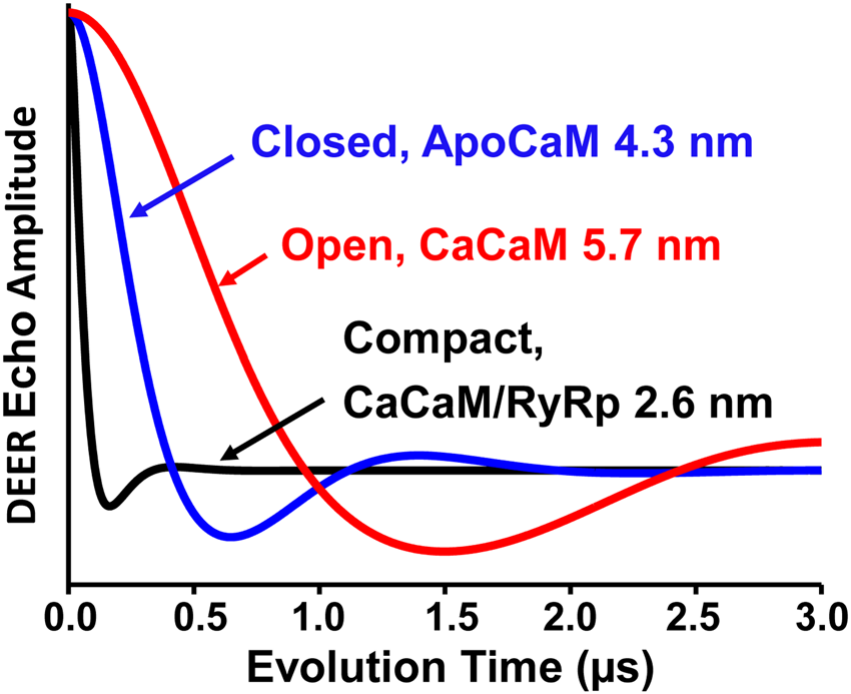
Simulations of DEER waveforms, based on distance predictions from structural models in Fig. 1, show that the three structural states of CaM should be clearly distinguished and resolved, with labeling sites at T34CS38C in the N lobe and R106CT110C in the C lobe. Each simulation assumed a Gaussian distance distribution with a width (FWHM) of 1.0 nm.

To determine optimal labeling sites for this study, DEER simulations were performed with BSL attached to different pairs of double-Cys mutations in the N- and C-lobes of CaM. The goal was to select a pair of BSL labeling sites to distinguish the previously proposed structural models: closed by NMR^8^, open by X-ray crystallography^4^, and compact by X-ray crystallography^15^. To achieve this, we required each BSL-labeled Cys residue to be on a stable α-helix and solvent-exposed, as shown in the proposed structural models (Fig. 1a–c). Figure 2 shows simulated DEER data for the optimized pair of BSL labeling sites in CaM, based on spin-labeled structural models in Fig. 1. The open model (5.7 nm) generates a DEER waveform with a slow decay and oscillation. The closed model (4.3 nm) generates a faster decay, and the compact model (2.6 nm) yields the fastest, consistent with a short distance on the lower end of DEER’s sensitivity range. All three structural states should be clearly distinguishable and resolved by DEER (Fig. 2).

### DEER resolves Ca-dependent structural states of CaM in solution

**Figure 3:**
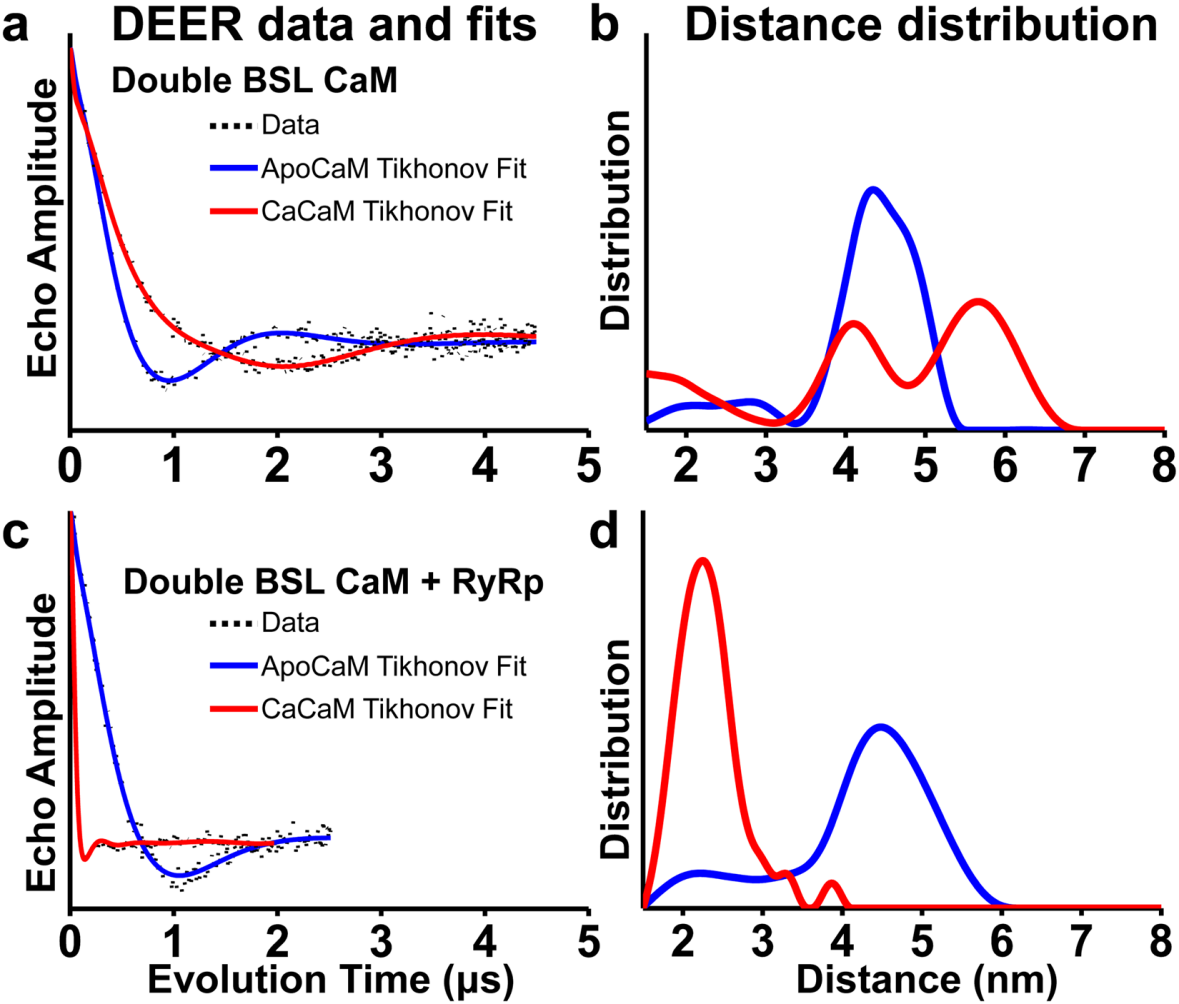
Intramolecular (lobe-to-lobe within CaM) DEER distance measurements resolving the Ca-dependent structural states (compact, closed and open) of double-labeled BSL-CaM, in the absence of Ca (blue, apo) or presence of saturating Ca (red). CaM was labeled at positions T34CS38C in the N-lobe and R106CT110C in the C-lobe. (**a,b**) DEER waveforms (black), best fits and distance distributions from Tikhonov regularization (blue/red) for double BSL-CaM (Fig. 1a,b). (**c,d**) DEER waveforms (black), best fits and distance distributions from Tikhonov regularization (blue/red) for the complex of double BSL-CaM and RyRp (Fig. 1c,d).

We measured four different conditions of double-labeled BSL-CaM with predicted lobe-to-lobe internitroxide distances in the range of 2–6 nm (Fig. 2). Figure 3a shows the results (data in black) of the background-corrected echo decay along with the corresponding best fits (blue for apoCaM and red for CaCaM). Figure 3b shows the distance distributions computed via Tikhonov regularization. ApoCaM (Fig. 3a, blue) yields a DEER waveform with a single predominant oscillation frequency, indicating a single major population. Calculation of the apoCaM distance distribution (Fig. 3b, blue) reveals the existence of one predominant population, having a distance centered between 4 and 5 nm, in excellent agreement with the predicted distance of 4.3 nm (Fig. 2, blue, from the NMR structure^8^). The fit also revealed a small fraction with a center distance between 2 and 3 nm, suggesting that apoCaM exists in equilibrium between the closed and compact states (2.6 nm, Fig. 2 black). The addition of Ca resulted in a DEER waveform with a much slower initial decay and longer period of oscillation (Fig. 3a, red), clearly indicating an increased distance. Analysis (Fig. 3b, red) confirms that the most populated component corresponds to a much longer distance (between 5 and 6 nm), in excellent agreement with the open structural state (5.7 nm, Fig. 2 red, from the crystal structure of CaCaM^4^). The width of this peak is relatively broad, perhaps from the central linker being highly mobile and allowing for the two lobe domains to tumble almost independently of one another as previously reported^3^. The width (and mole fraction) of this portion of the distribution is tightly coupled to choice of background component and is therefore less certain than the distance (Fig. S2). Furthermore, two additional structural states are clearly resolved by DEER, one at ~4 nm, in good agreement with the predicted closed state (4.3 nm, Fig. 2 blue); and another ~2 nm (near the short distance limit of DEER), in good agreement with the predicted compact state (2.6 nm, Fig. 2, black). *Thus DEER reveals for the first time that in the presence of Ca, CaM exists in equilibrium among all three previously detected structural states*. This is the first evidence for CaCaM having a significant population in the closed state (previously observed only for the NMR structure of apoCaM^4^), or the compact state (previously observed only once for the crystal structure of CaCaM alone^6^ and CaM bound to a target peptide such as RyRp^15^**)**.

### RyRp binding to CaM occurs through a conformational selection mechanism

The above results strongly support a *conformational selection* mechanism, in which CaM binding to a target protein occurs through “selection” of pre-existing CaM conformations^34^. To test this hypothesis further, with respect to the CaM/RyR interaction, we performed DEER measurements on double labeled BSL-CaM bound to unlabeled RyRp (Fig. 3c,d). In the absence of Ca, the DEER waveform is unaffected by RyRp binding (Fig. 3c, blue). Analysis (Fig. 3d, blue) confirmed that apoCaM remains predominantly in the closed state (4–5 nm), consistent with the closed state observed in the absence of RyRp (4–5 nm, Fig. 3b, blue). A much more dramatic effect of RyRp is observed in the presence of Ca: the DEER waveform shows a much faster initial decay (Fig. 3c red) than in the absence of RyRp (Fig. 3a, red), indicating a substantial decrease in the interlobe distance. Indeed, the observed waveform is in excellent agreement with that predicted (Fig. 2 black, 2.6 nm) by the compact state of the CaCaM/RyRp complex (Fig. 1c). Thus our DEER results clearly support a conformational selection mechanism, in which the addition of RyRp to CaCaM shifts the structural equilibrium toward the compact state, with little to no effect on apoCaM.

### Ca Stabilizes the compact state by increasing binding of the CaM N-lobe to RyRp

**Figure 4:**
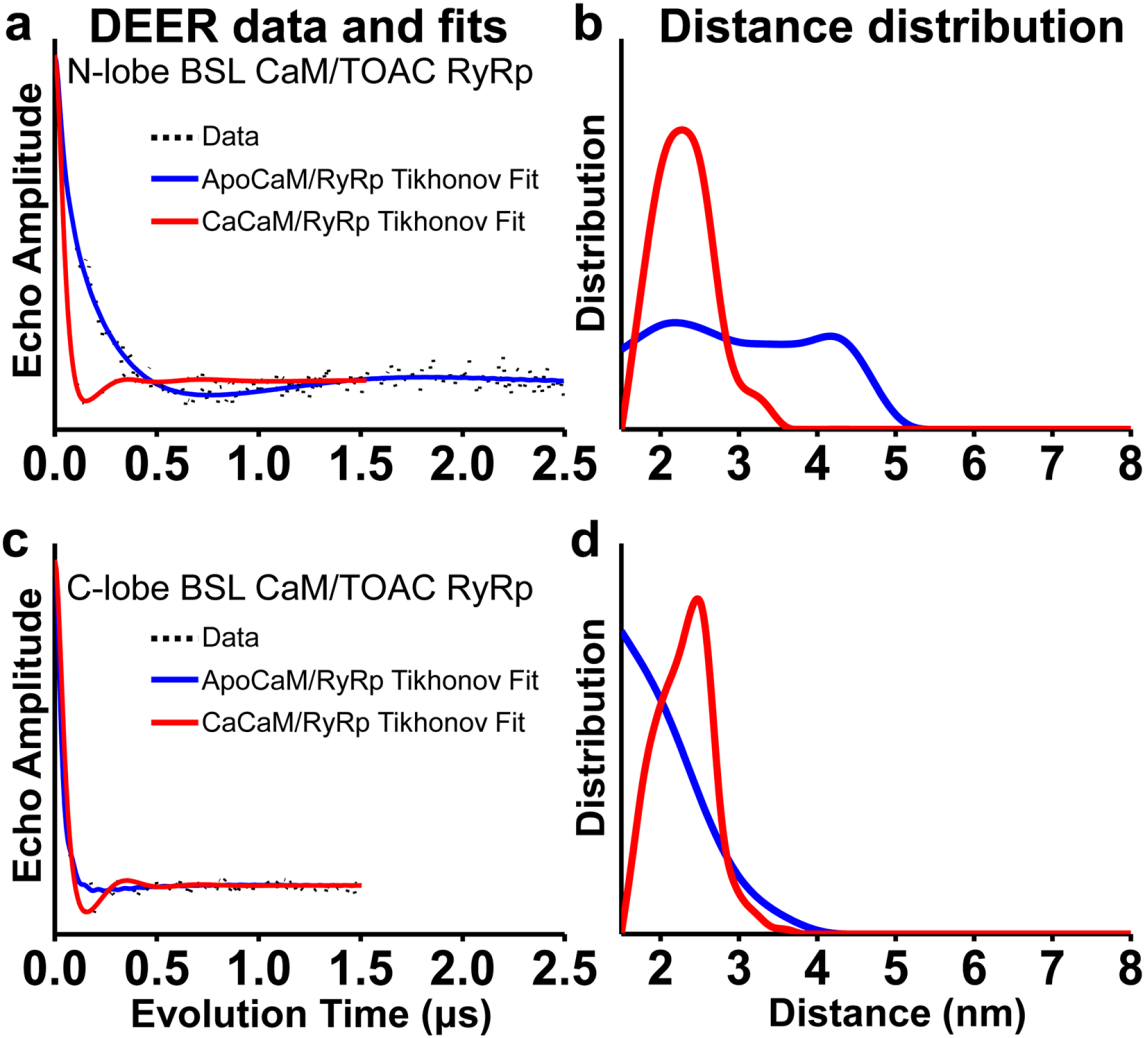
Intermolecular (between CaM and RyRp) DEER distance measurements resolving the Ca-dependent structural states of the CaM/RyRp complex, in the absence of Ca (blue, apo, Fig. 1f) or presence of saturating Ca (red, Ca, Fig. 1e). Distance measurements were made between 5-TOAC-RyRp and N-lobe BSL-CaM (**a,b**), and 5-TOAC-RyRp and C-lobe BSL-CaM (**c,d**).

To further characterize the conformational selection mechanism of CaM binding to RyRp, we used DEER and dipolar CW-EPR to measure the Ca-dependent distance distribution between BSL on the N-lobe and TOAC on RyRp. Figure 4a shows the results (data in black) of the background-corrected DEER echo decays along with the corresponding best fits (blue for apoCaM and red for CaCaM). Inspection of the data for apoCaM reveals a DEER waveform with dampened oscillations, consistent with a broad distance distribution and thus disorder. Indeed, determination of the distance distribution reveals the presence of two overlapping populations with the center distances of one between 2 and 3 nm, and the other between 4 and 5 nm (Fig. 4b, blue). The addition of Ca resulted in a much faster initial decay and a shorter period of oscillation (Fig. 4a, red), indicating decreased distance and increased order. Analysis revealed one predominant compact state with a center distance between 2 and 3 nm (Fig. 4b, red), consistent with the shorter of the two populations observed in the absence of Ca (Fig. 4b, blue). Thus, supporting the conformational selection model, Ca stabilizes the pre-existing compact state, probably by increased binding of the N-lobe of CaM to RyRp.

### C-lobe of CaM binds to RyRp independent of Ca

We performed analogous experiments with BSL on the C-lobe of CaM and TOAC on RyRp (Fig. 4c and d). In contrast with the N-lobe experiments, the effect of Ca is small, with rapid decays indicating short distances in both cases, although there is more disorder in the absence of Ca. Thus, our DEER results are consistent with the hypothesis that the C-lobe of CaM remains bound to RyRp independent of Ca^25,35,36^, and the largest effect of Ca is mainly to increase the order of the C-lobe with respect to RyRp.

### Dipolar CW EPR confirms the existence of populations with short interprobe distances

Intermolecular DEER measurements between BSL-CaM and TOAC-RyRp resulted in populations near or below the 2.0 nm limit of DEER sensitivity (Fig. 4b,d). To evaluate whether there were significant populations below this limit, we performed dipolar CW EPR on all BSL-CaM/TOAC-RyRp samples (Fig. S7). N-lobe BSL-CaM in complex with TOAC-RyRp reveals minimal broadening in the dipolar spectrum in the presence of Ca (Fig. S7a, red), while no significant broadening was observed in its absence (Fig. S7a, blue). With BSL on the C-lobe, minimal broadening was observed in the absence of Ca but not in its presence (Fig. S7d), and no distances substantially shorter than 2 nm were detected. These results are entirely consistent with the DEER results discussed above, indicating that DEER accurately captured all relevant structural states in this system.

## Discussion

This is the first report in which two different stereospecific spin labels have been used together in a single EPR study. We used peptide synthesis and mutagenesis to introduce TOAC and BSL, respectively. We demonstrated that these spin labels, which offer *rigid and specific attachment relative to the protein backbone*, are powerful probes to detect and resolve structural states. TOAC reflects backbone dynamics directly^21,23,25,37^. BSL is an alternative to TOAC that can be applied to larger proteins, as long as Cys residues can be engineered at i and i + 4 on an α-helix^27–29,38^. These labels provide a substantial advantage over conventional methods of site-directed spin labeling, in which the attachment of probes to single Cys side chains leave several flexible bonds between the α-carbon and the nitroxide group, thus leaving considerable ambiguity about the interpretation of the spectra.

**Figure 5:**
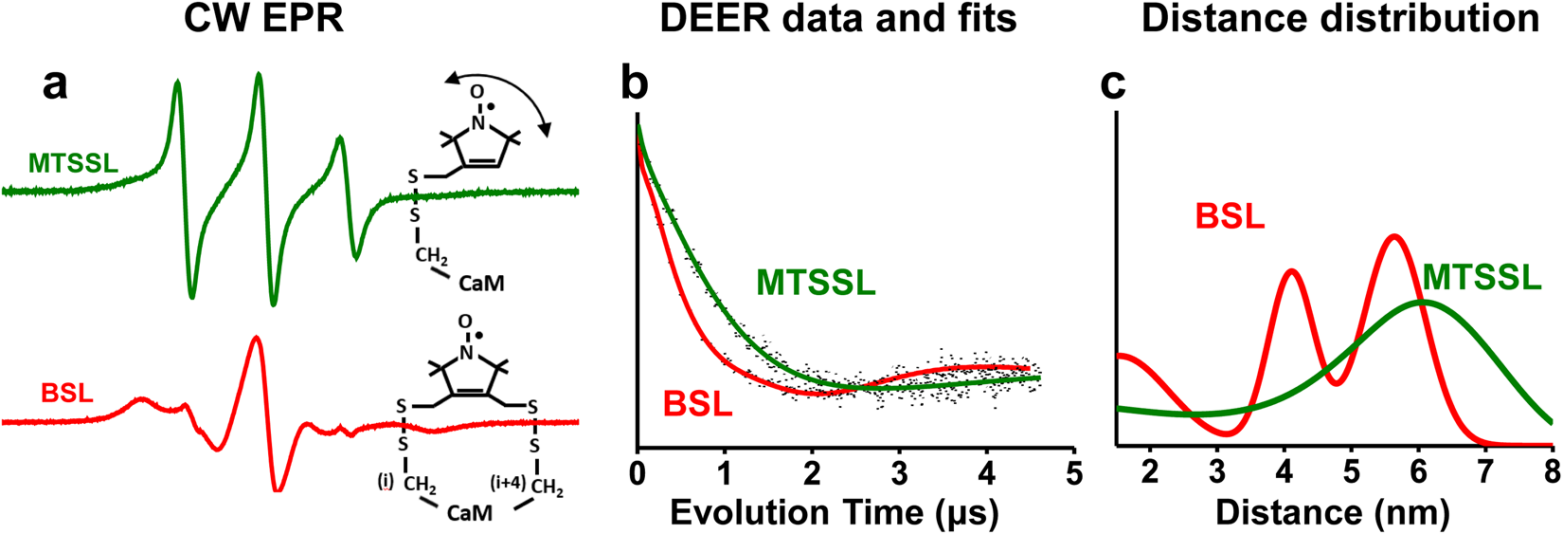
Comparison of MTSSL (green) and BSL (red) in detecting backbone dynamics and distance distributions within CaM. (**a**) CW EPR spectra at 4 °C, showing that BSL (labeled at position T34CS38C) is strongly immobilized (broad spectrum) on CaM, accurately detecting backbone dynamics, while MTSSL (labeled at position T34C) is weakly immobilized, indicating spin label flexibility (double-headed arrow). (**b**) Background-corrected DEER echo decay of double BSL-CaCaM (T34CS38C-R106CT110C) and double MTSSL-CaCaM (T34C-T110C). (**c**) DEER distance distributions demonstrating the resolving power of BSL. MTSSL reveals two unresolved, broad distance distributions (green) while BSL reveals three distinct distance populations (red). CW EPR spectra in A were obtained with a 120 G scan width, then normalized to unit spin concentration by dividing by the double integral.

These points are illustrated clearly in Fig. 5, which compares the CW EPR and DEER results for MTSSL-CaM with those of BSL-CaM labeled at the same position. The BSL CW spectrum is much broader than that of MTSSL, indicating greatly reduced nanosecond probe motion and disorder (Fig. 5a). The spectrum of BSL-CaM indicates a rotational correlation time of 8 ± 2 ns, consistent with the global tumbling of CaM^39^ and the rigid attachment of the probe to the peptide backbone. The spectrum of MTSSL-CaM indicates a rotational correlation time less than 1 ns^39^, consistent with flexible attachment of the probe. The DEER waveform of BSL-CaM shows clear oscillations (Fig. 5b, red) and resolves three distinct structural states (Fig. 5c, red), while MTSSL shows no discernable oscillation (Fig. 5b, green) and yields a broad distance distribution (Fig. 5c, green). Thus BSL is clearly superior to MTSSL for accurately reflecting the dynamics and locations of backbone atoms, and resolving the structural states of the complex.

**Figure 6:**
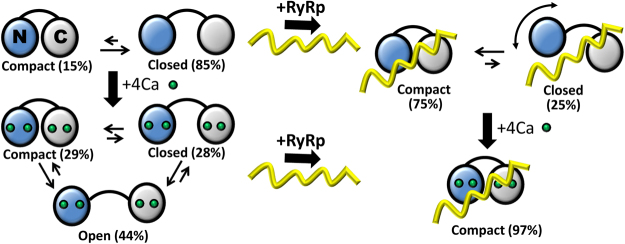
Schematic of structural models based on DEER distance measurements (Figs 3 and 4). Blue, N-lobe of CaM; gray, C-lobe of CaM; yellow, RyRp; green, Ca ion. In the absence of RyRp and Ca (upper left) CaM is primarily in the closed structural state. Ca binding to CaM (bottom left) populates all three states (open, closed, compact), with the open state predominant. In the presence of RyRp and the absence of Ca (upper right), the complex is in dynamic equilibrium between the compact state, and a closed-like state with the N-lobe potentially being highly mobile and not bound to RyRp. Ca binding to CaM (bottom right) shifts the complex almost completely to the compact state by increasing binding of the N-lobe to RyRp.

Figure 6 illustrates a model for the conformational selection mechanism of CaM binding to RyRp, as supported by our intraCaM DEER results (Fig. 3) and previous studies of CaM binding to other target peptides^40–42^. In addition, we report for the first time direct high-resolution structural measurements on the apoCaM/RyRp complex (Fig. 3c,d, blue; Fig. 4 blue), allowing us to propose a structural model for this complex (Fig. 1d,f), providing new structural insight into the Ca-dependent interaction of CaM and RyR.

As the resultant distributions reveal distinct subpopulations, we further parameterized the waveforms by fitting the background-corrected echo decays to a sum of Gaussians model, in order to extract mole fractions and ascertain the uncertainty of the fit parameters, as described in *Materials and Methods* and in *Supplemental Information* (Figs S2,S3 and S5; Tables S1 and S2). While quantitative analyses from Gaussian modeling may yield numerical values with high uncertainty, especially for distances near the sensitivity limits of DEER, they do nevertheless provide valuable insight into the observed population changes caused by Ca and RyRp binding. In addition to uncertainty found in the error surface of the fit (Fig. S5 and Tables S1, S2) and the appropriateness of model choice, several experimental factors warrant interpreting both the parameterized and Tikhonov distance distributions with caution. In the case of short distances (≤3 nm), limits in our excitation bandwidth result in errors in population center and fraction due to incomplete excitation of short distance spin-pairs^43^. For longer distances, experimental constraints limit our maximal evolution time (which determines the maximum resolvable distance^32^, see Figs S2–S4), allowing for the potential of un-resolved populations and errors in population width and therefore fraction near the sensitivity limit.

In the absence of RyRp and Ca, apoCaM exists in equilibrium between a predominantly closed state (85%, consistent with the NMR structure^8^) and a weakly-populated compact state (15%) (Fig. 6, top left). Ca shifts this distribution to a more complex one (Fig. 6, bottom left), in which CaCaM exists in structural equilibrium among three distinct structural states: compact (29%), closed (28%) and open (44%). CaCaM has a diverse array of binding modes to over 300 targets;^44^ probably facilitated by the complex conformational equilibrium reported here. RyRp binding to CaCaM induced an almost complete structural equilibrium shift toward a compact structure (Fig. 6, bottom right) consistent with the X-ray crystal structure CaCaM/RyRp complex^15^. Figure 6 (top right), illustrates the new proposed structural model of the apoCaM/RyRp complex, which is primarily in equilibrium between compact (75%) and closed (25%) structural states, while addition of Ca shifts the equilibrium almost completely toward the compact state (97%). The compact state of CaCaM/RyRp was previously reported by X-ray crystallography^15^, but here, we show directly for the first time the structural transition from the compact (75%) and closed (25%) states in the absence of Ca to a single compact state (97%) of the CaM/RyRp complex upon addition of Ca.

At first glance, the addition of RyRp has no effect on the lobe-lobe distance in apoCaM (Fig. 3b and d, blue; Table S1), suggesting that RyRp may not be binding CaM in the absence of Ca. However, two lines of evidence indicate that there is at least partial binding between apoCaM and RyRp, and Ca is required for maximum binding, as supported by our DEER measurements. First, binding has been detected in the absence of Ca by several investigations at a lower affinity than in the presence of Ca^35,36,45^. Second, we have previously shown by CW EPR that TOAC-labeled RyRp was only partially immobilized by apoCaM while CaCaM led to complete immobilization of the peptide^25^. These observations led us to try to detect the presence of binding, although potentially weak, by labeling the lobes of CaM individually with BSL and the RyRp with TOAC through peptide synthesis. In this system, if there were no binding in the absence of Ca, we would not be able to measure any inter-protein DEER distances (Fig. 4, blue), therefore at least some fraction of RyRp is bound to apoCaM. Thus, it is possible that a large fraction of the double BSL apoCaM/RyRp sample is uncomplexed proteins (giving it a distance distribution similar to apoCaM alone), and the DEER signal coming from the BSL N-lobe CaM/TOAC RyRp sample is from a smaller bound fraction (with the labeled CaM and RyRp not in complex contributing merely to the background signal).

Previous investigations by us^25^ and others^35,45,46^, suggest that the N-lobe of CaM is involved in tight binding of CaM to RyRp, and this is important for regulation of RyR^36^. Our DEER distance measurement between the N-lobe of CaM and RyRp revealed the flexibility and structural heterogeneity of this complex, with two broad distance populations in the absence of Ca (Fig. 4b, blue and Table S2); the major population (75%) corresponds to the compact state. The longer distance population is too long to be explained by the compact state of CaM, so this population probably represents a state where the N-lobe is not bound tightly to RyRp. Addition of Ca shifted the equilibrium to one predominant population with a short distance (Fig. 4b, red and Table S2), indicating complete binding of the N-lobe to RyRp in a compact state. In contrast, distance measurement between the C-lobe of CaM and RyRp indicate that the C-lobe is tightly bound to RyRp independent of Ca (Fig. 4d), with Ca only slightly decreasing the disorder (Table S2). These results are consistent with our previous report in which complete immobilization of the C-terminal region of TOAC-labeled RyRp (by CaM N-lobe) was Ca-dependent, whereas the N-terminal region of RyRp was completely immobilized (by the C-lobe of CaM) independent of Ca^25^. Thus, we present further structural evidence that supports the hypothesis that CaM functions as a subunit of RyR through binding of the C-lobe, and complete interaction of the N-lobe of CaM (in response to increased cytosolic Ca levels) is responsible for maximum inhibition of RyR.

## Conclusions

We report a unique approach utilizing a combination of BSL, TOAC, and DEER, to resolve and quantitate the structural states of CaM as affected by Ca and RyRp binding. Our results directly support a conformational selection model of CaM binding to RyRp, which occurs by RyRp shifting the structural equilibrium of CaM toward pre-existing states. We were also able to directly detect and resolve structural states of CaM/RyRp even in the absence of Ca with sub-nanometer precision, while such high-resolution information is not available from X-ray crystallography or NMR. We detected structural changes in each CaM lobe with respect to RyRp binding, and showed that the N-lobe/RyRp interaction was much more sensitive to Ca than the C-lobe, providing unique structural insight into the mechanism of Ca-dependent CaM-mediated RyR regulation. Finally, we have demonstrated the power of TOAC and BSL in measurement of protein backbone rotational dynamics and interspin distance (Fig. 5). This establishes the power of DEER spectroscopy with stereospecific spin labels as a tool that complements conventional structural techniques. This approach will be powerful in future studies of CaM and other Ca-binding proteins, with important implications for molecular pathology and therapeutic development for muscle disorders.

## Materials and Methods

### Computational approach

The Discovery Studio Visualizer software (windows version 2.5.5.9350) was used to optimize BSL-CaM labeling sites in the N-lobe and C-lobe. For this purpose, PDB files (Fig. 1a. 1CFD, B. 1CLL, C. 2BCX) were individually opened by the software. In the second step two amino acids in the N-lobe and C- lobe at i and i +4 positions were chosen to be mutated to Cys. In the modified PDB files, BSL was attached in the N-lobe (T34CS38C) and C-lobe (R106CT110C). The distances from nitrogen to nitrogen between the attached BSL labels in the N-lobe and C-lobe were determined (Fig. 1a–d). The shorter distance from the singly labeled BSL- CaM mutant (T34CS38C) to 5-TOAC-RyRp (TOAC substituted for Ala5) was accomplished in a similar way, by using the modified PDB file 2BCX (Fig. 1e,f). The estimated distances were then used to simulate the expected DEER waveforms (Fig. 2) using WACY, an in-house laboratory developed EPR software (Edmund Howard).

### CaM mutagenesis and purification

Tetra-Cys CaM was engineered containing two Cys in the N-lobe (T34CS38C) and C-lobe (R106CT110C) followed by cross linking with BSL to measure intra-molecular distances by DEER. Di-Cys CaM mutants (T34CS38C or R106CT110C) were also expressed and purified for experiments involving inter-protein distance measurement with TOAC labeled RyRp. In detail, recombinant human CaM was expressed in *Escherichia coli* using the pET-7 vector^47^. Mutations targeting the Cys labeling sites were introduced using the QuikChange mutagenesis kit (Stratagene, La Jolla, CA), and the mutation was verified by DNA sequencing. Following induction with isopropyl β-D-1-thiogalactopyraoside, di- and tetra-Cys-CaM were purified via phenyl-Sepharose chromatography^48^. Protein concentrations were determined by the bicinchoninic acid procedure (Pierce, Rockford, IL) using bovine brain CaM as the standard^49^.

### BSL-CaM spin labeling

BSL-CaM spin labeling was accomplished by first reducing the protein with 5 mM DTT for one hour at room temperature. DTT was removed using Zeba Spin desalting columns (Thermo Scientific) and the reduced CaM sample then incubated with 4 × molar excess BSL (Toronto Research Chemicals) at 4 °C overnight. Excess BSL was removed and the protein was exchanged into EPR buffer (20 mM MOPS, 5 mM Ca or 5 mM EGTA, pH 7.4) using a Zeba Spin desalting column. Spin counting results showed that ≥80 of BSL-CaM were labeled.

### Synthesis of TOAC-RyRp and RyRp

RyRp and TOAC-RyRp (with TOAC substituted for Ala5) were synthesized by solid-phase peptide synthesis (SPPS) as previously described^25^. Spin counting results showed that ≥60 of TOAC-RyRp were labeled. Cys22 was replaced with α-amino-n-butyric acid (Abu) to prevent disulfide exchange with BSL labeled CaM. EPR studies of BSL labeled CaM with WT-RyRp (Cys 22 instead of Abu) caused the appearance of a highly mobile spectral component, corresponding to free spin label. BSL labeled CaM showed only 41% spin labeling efficiency. The most likely interpretation of the free BSL label is that the Cys residue of WT-RyRp participates in disulfide exchange with BSL-CaM as previously reported^50^. BSL is a sulfhydryl-reactive cross linker and linked by disulfide bonds to the –SH peptide side chain. The reaction between pairs of free or reduced sulfhydryl groups (-SH) is selective and precise. To control the number of free –SH groups we replaced Cys22 with Abu.

### CD spectroscopy

CD spectra were acquired at 22 °C with a JASCO J-815 spectrophotometer, scanning from 190 nm to 260 nm with a scan rate of 50 nm/min, 0.1 nm bandwidth. Spectra were signal-averaged five times and baseline subtracted. Reported spectra are expressed as mean residue ellipticity, [θ]. Linear combinations of α-helix, β-sheet and random coil bases spectra were used to determine secondary contributions from fits to acquired CD spectra^51,52^. Samples for CD consisted of 20 μM CaM, BSL-CaM or double BSL-CaM in 10 mM Na_2_HPO_4_. pH 7.4, loaded into quartz cuvettes with a path length of 0.1 cm. We previously reported that TOAC labeling of RyRp does not significantly alter its secondary structure and how it responds to the alpha-helix inducer TFE^25^. We performed CD on BSL labeled CaM mutants and show that single (T34CS38C) and double labeled BSL-CaM (T34CS38CR106CT110C) does not significantly alter the secondary structure compared to wild-type CaM (Fig. S1). The linear fit of the CD spectrum yielded 90.3% α-helix for WT-CaM, 89.8% for single BSL-CaM and 88.1% for double BSL-CaM (Fig. S1).

### EPR spectroscopy and data analysis

We performed DEER to measure distances from 2 to 6 nm, and dipolar CW-EPR for distances from 0.5 to 2 nm. DEER signals were acquired with an Elexsys E580 spectrometer (Bruker) operating at Q-band (34 GHz) equipped with a EN5107 resonator using a four-pulse DEER sequence^32^ with a 12 ns π/2 pulse and a 24 ns ELDOR pulse. The spacing between the echo forming π/2 and π pulses was incremented in 8 steps of 16 ns to average out D_2_O artifacts^32^. The pump frequency was assigned to the maximum of the nitroxide absorption spectrum and the observe frequency placed at a 24 Gauss higher magnetic field strength on the field swept absorption spectrum. Data was acquired at 65 K and lasted 16–24 hours. All DEER samples contained 90–100 µM CaM in 20 mM MOPS, pH 7.4, 95% D_2_O. 10% glycerol was added for cryoprotection. Samples containing RyRp had a ratio of 1.25:1 (peptide:CaM). DEER waveforms were analyzed using custom software written in Mathematica based on DeerAnalysis^53^ and DEFit^54^ (github.com/thompsar). Background corrected waveforms were analyzed using Tikhonov regularization, with choice of smoothing parameter informed by both the l-curve and leave one out cross validation (LOOCV) criteria^55^. After stable populations were identified via a systematic variation of the region fit for the background component (similar to DeerAnalysis’ validation tool), highly unstable populations that were well separated from the primary distribution (and beyond the sensitivity range afforded by acquired evolution time) were suppressed by repeating background correction with the appropriate spectral components included as a correction to the initial homogeneous background model, followed again by Tikhonov regularization. The resultant filtered Tikhonov distribution (which showed no distortion beyond the suppression of the unstable components) was then used as seed for a Monte Carlo fit of the waveform using a sum of Gaussians model, ρ_j_(R), determining population centers, widths and mole fractions as well as their respective uncertainties with respect to the error surface of the fit (Fig. S5):

1 $${\rho }_{{\rm{j}}}(R)=\frac{1}{{\sigma }_{{\rm{j}}}\sqrt{2\pi }}\,\exp \,(\frac{-{[R-{R}_{{\rm{j}}}]}^{2}}{2{\sigma }_{{\rm{j}}}^{2}})$$

2 $${\sigma }_{{\rm{j}}}={{\rm{FWHM}}}_{{\rm{j}}}/(2\sqrt{2\,\mathrm{ln}\,2})$$

where σ is the standard deviation and FWHM is the full width at half maximum of the distribution. The minimal number of Gaussians necessary to produce a satisfactory fit was determined by both a comparison of RMSDs with the Tikhonov fit as well as using Bayesian information criterion^56^, which in our experience performs better than the Akaike information criterion^57^ at picking the most parsimonious model.

For dipolar CW-EPR measurements of BSL-CaM to 5-TOAC-RyRp, samples were prepared in 20 mM MOPS, pH 7.4, 95% D_2_O at a concentration of 90 μM of spin-labeled protein. All CW-EPR spectra were recorded at X-band (9.6 GHz) with a Bruker E500 Elexsys spectrometer equipped with an ER 4122 SHQ resonator. For dipolar EPR spectra acquisition, temperature was maintained at 200 K with a quartz dewar insert and nitrogen gas flow temperature controller. All frozen spectra were acquired using 0.6 mW power to prevent spin saturation, and 200 G sweep width. All spectra were baseline correct then normalized to the double integral prior to analysis. For determination of spin-spin distance from dipolar CW-EPR spectra of BSL-CaM/5-TOAC-RyRp, spectral simulation and least-square fits were performed as previously described (Fig. S6)^58^. Essentially, experimental spectra were fit to a model corresponding to a sum of Gaussian distance distributions. CW EPR spectra of BSL-CaM at 4 °C were analyzed to determine the rotational correlation time,

3 $${\tau }_{{\rm{R}}}=a{[1-({T}_{\parallel }\text{'}/{T}_{\parallel })]}^{{\rm{b}}},$$

where a = 0.54 ns, b = −1.36, *T*_*||*_ʹ = splitting between the outer extrema, and *T*_*||*_ = the rigid-limit value of *T*_*||*_ʹ^59^. Each spectrum was analyzed to determine the order parameters S,

4 $${\rm{S}}=({T}_{||}\text{'}-{T}_{0})/({T}_{||}\text{'}-{T}_{0}),$$

where *T*_||_′ = splitting between the outer extrema, *T*_||_ = the rigid-limit value of *T*_||_′ and *T*_0_ = the fast-limit value of *T*_||_′. Results indicate no significant detectable differences in CW EPR dynamics between the N- and C-lobes (Table S3).

## Electronic supplementary material

Supplemental Information
