# Supplementary material for: Structural dynamics of calmodulin-ryanodine receptor interactions: electron paramagnetic resonance using stereospecific spin labels
Source: Sci Rep. 2018 Jul 16;8:10681. doi: 10.1038/s41598-018-29064-8 (PMC6048129; doi:10.1038/s41598-018-29064-8)
Supplement: Supplementary file 1 — Supplemental Information [file 41598_2018_29064_MOESM1_ESM.pdf]

SUPPLEMENTAL INFORMATION

Structural dynamics of calmodulin-ryanodine receptor interactions: electron paramagnetic resonance using stereospecific spin labels

Cheng Her, Andrew R. Thompson, Christine B. Karim, and David D. Thomas

Department of Biochemistry, Molecular Biology and Biophysics, University of Minnesota, Minneapolis, Minnesota 55455

Supplemental Figures and Tables

a

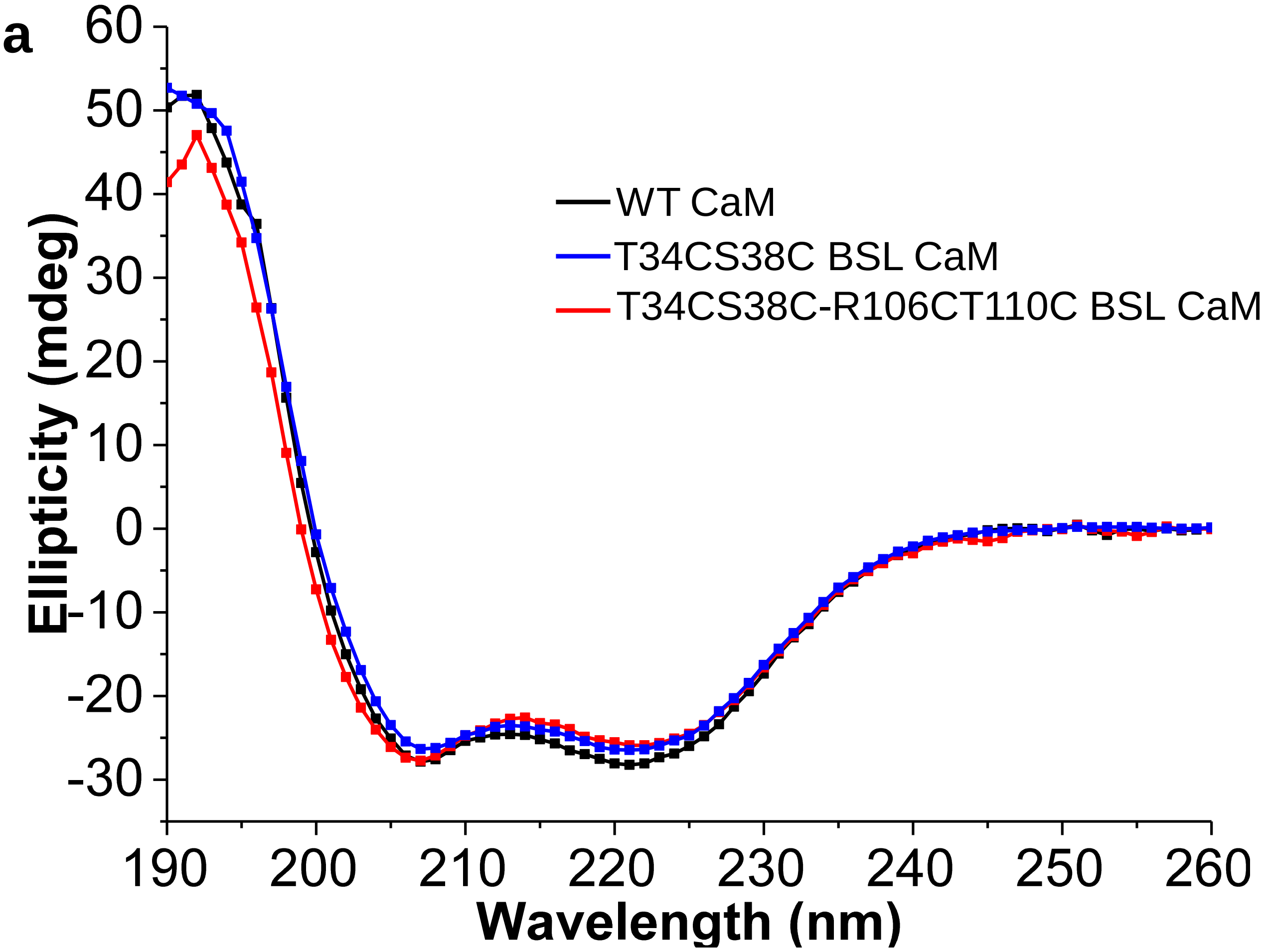

b

Protein	WT unlabeled CaM	T34CS38C BSL CaM	T34CS38C R106CT110C BSL CaM
% α-helix	90.3 ± 0.5 %	89.8 ± 0.6 %	88.1 ± 1.0 %

Fig. S1. (**a**) CD spectra of WT CaM (black), single BSL-CaM (blue) and double BSL-CaM (red). (**b**) Percent α-helix were determined from data simulation and fitting assuming a linear combination of α-helix, β-sheet and random coil. In either case, BSL labeling did not significantly disrupt the secondary structure of CaM.

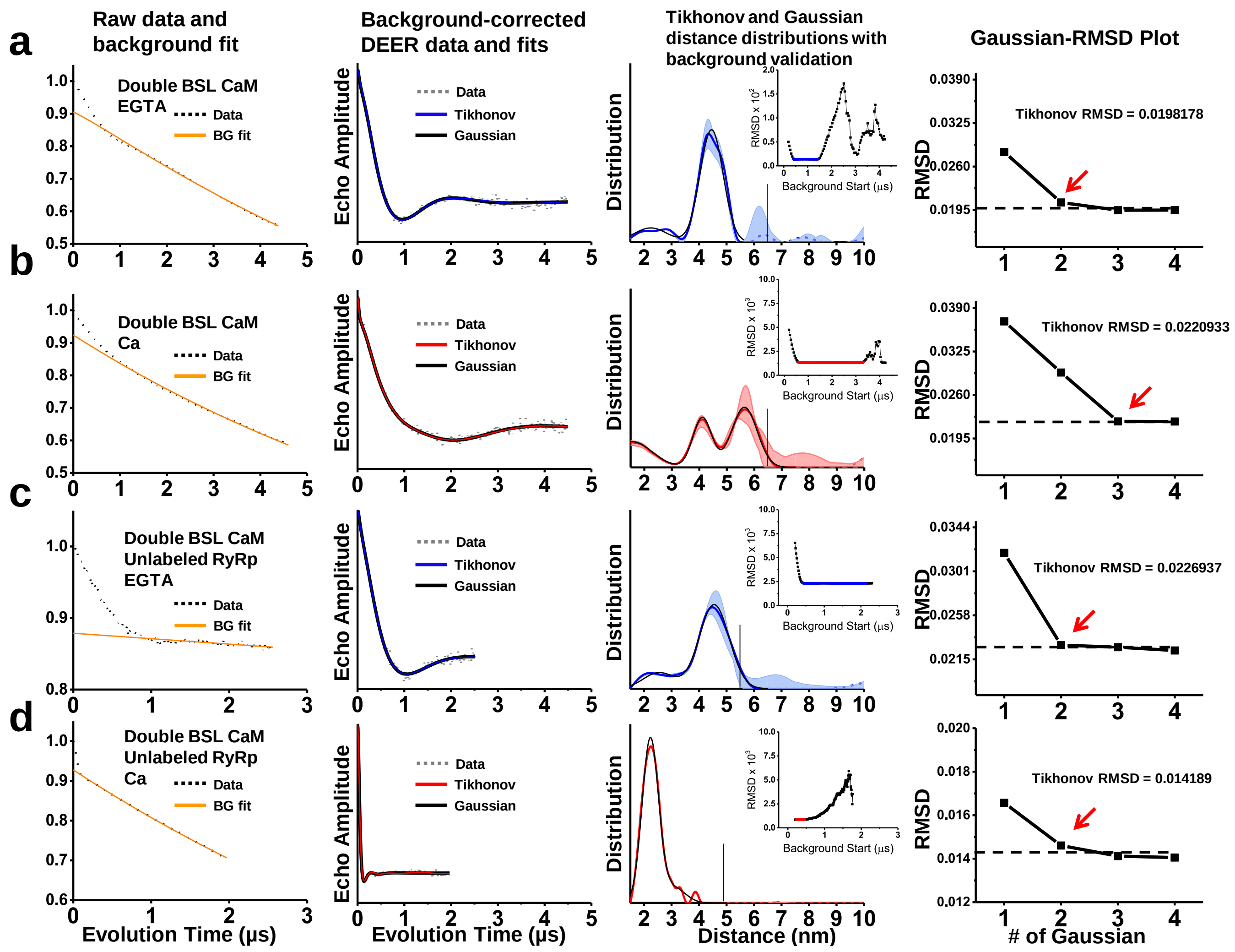

Fig. S2. Analysis of double BSL CaM DEER in the absence of Ca (**a**), presence of saturating Ca (**b**), presence of unlabeled RyRp and no Ca (**c**), and presence of unlabeled RyRp and saturating Ca (**d**). *First column* shows the raw data (black dots) and background fit (orange). *Second column* shows the background-corrected data (gray dots, using the shown homogenous background model augmented with the selected long range distance components), Tikhonov fits (red/blue), and best Gaussian fits (black). *Third column* shows Tikhonov distance distribution (red/blue), 95% CIs from background validation (shaded), and Monte Carlo sum of Gaussians model (black). Unstable populations (dashed) were suppressed from the primary distribution (solid) by a via modification of the background model as described with no distortion to the primary distribution shown here and in Figs. 2 and 3 in the manuscript. The primary Tikhonov distribution was then used as a seed for a Monte Carlo fit of the waveform using a sum of Gaussians model shown here (black). Insets show range of background validation RMSD with selections in red/blue. The vertical line indicates the maximum reliable mean detectible distance given the waveform's evolution time (Ref. 31 in main text). *Fourth column* shows the refinement of the Gaussian model, with the red arrow in the RMSD-Gaussian plot showing the number of Gaussians sufficient to fit the data (n), at which n + 1 did not significantly improve the fit, informed by Bayesian information criterion. See Materials and Methods for more details.

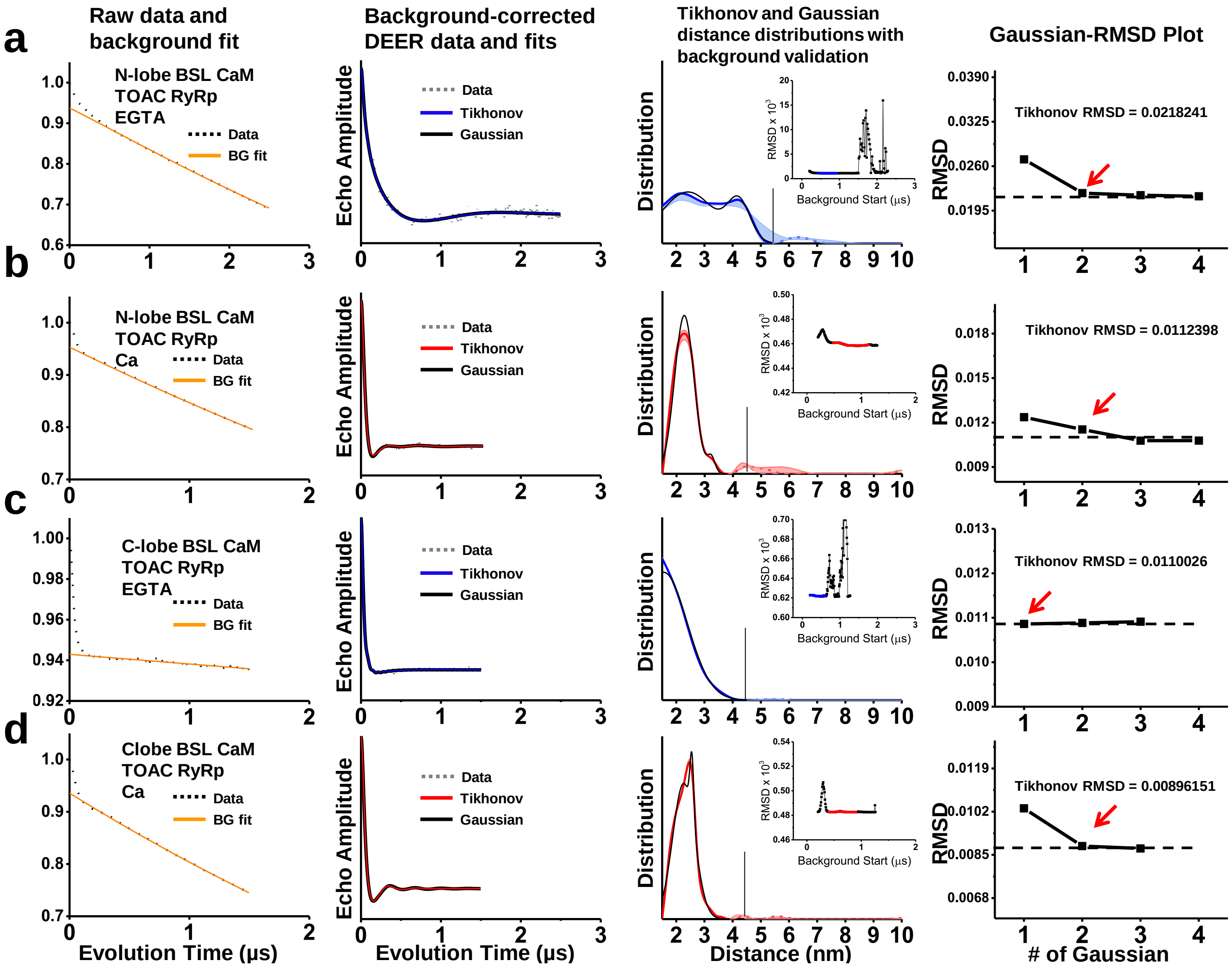

Fig. S3. Analysis of BSL-CaM/TOAC-RyRp DEER. Distances measured were between N-lobe BSL CaM and TOAC RyRp in the absence of Ca (**a**), N-lobe BSL CaM and TOAC RyRp in the presence of saturating Ca (**b**), C-lobe BSL CaM and TOAC RyRp in the absence of Ca (**c**), and C-lobe BSL CaM and TOAC RyRp in the presence of saturating Ca (**d**). *First column* shows the raw data (black dots) and background fit (orange). *Second column* shows the background-corrected data (gray dots, using the shown homogenous background model augmented with the selected long range distance components), Tikhonov fits (red/blue), and best Gaussian fits (black). *Third column* shows Tikhonov distance distribution (red/blue), 95% CIs from background validation (shaded), and Monte Carlo sum of Gaussians model (black). Unstable populations (dashed) were suppressed from the primary distribution (solid) by a via modification of the background model as described with no distortion to the primary distribution shown here and in Figs. 2 and 3 in the manuscript. The primary Tikhonov distribution was then used as a seed for a Monte Carlo fit of the waveform using a sum of Gaussians model shown here (black). Insets show range of background validation RMSD with selections in red/blue. The vertical line indicates the maximum reliable mean detectible distance given the waveform's evolution time (Ref. 31 in main text). *Fourth column* shows the refinement of the Gaussian model, with the red arrow in the RMSD-Gaussian plot showing the number of Gaussians sufficient to fit the data (n), at which n + 1 did not significantly improve the fit, informed by Bayesian information criterion. See Materials and Methods for more details.

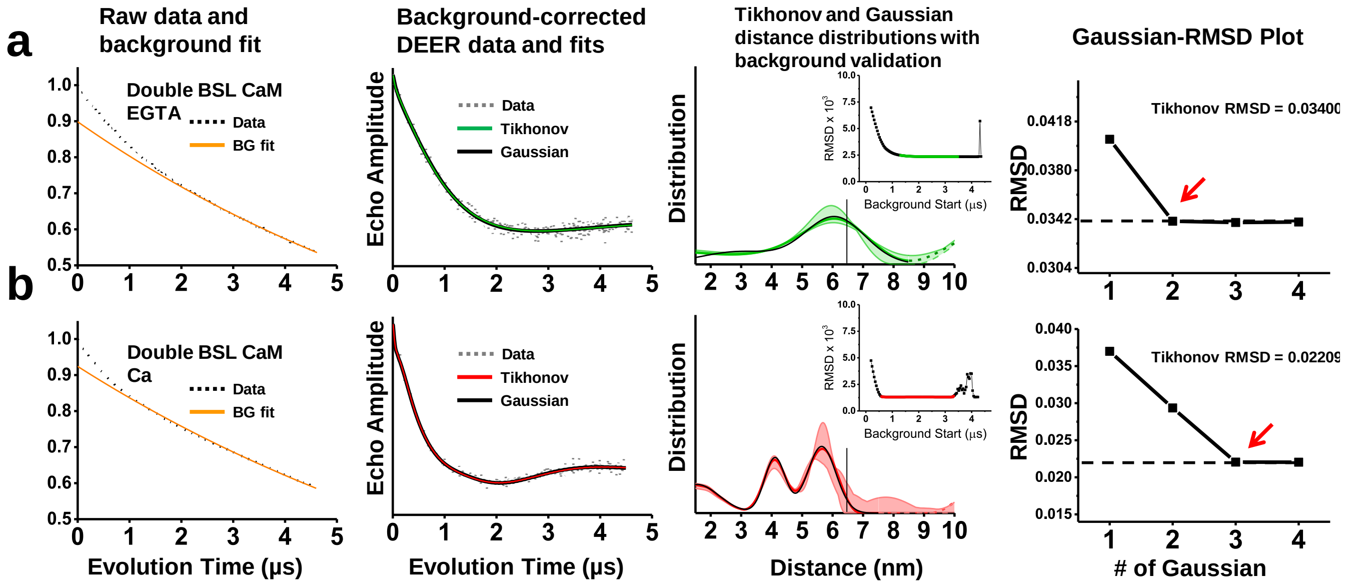

Fig. S4. Analysis of DEER distances measurement in the presence of saturating Ca for double MTSSL CaM (**a**) and double BSL CaM (**b**). *First column* shows the raw data (black dots) and background fit (orange). *Second column* shows the background-corrected data (gray dots, using the shown homogenous background model augmented with the selected long range distance components), Tikhonov fits (green/red), and best Gaussian fits (black). *Third column* shows Tikhonov distance distribution (green/red), 95% CIs from background validation (shaded), and Monte Carlo sum of Gaussians model (black). Unstable populations (dashed) were suppressed from the primary distribution (solid) by a via modification of the background model as described with no distortion to the primary distribution shown here and in Figs. 2 and 3 in the manuscript. The primary Tikhonov distribution was then used as a seed for a Monte Carlo fit of the waveform using a sum of Gaussians model shown here (black). Insets show range of background validation RMSD with selections in red/blue. The vertical line indicates the maximum reliable mean detectible distance given the waveform's evolution time (Ref. 31 in main text). *Fourth column* shows the refinement of the Gaussian model, with the red arrow in the RMSD-Gaussian plot showing the number of Gaussians sufficient to fit the data (n), at which n + 1 did not significantly improve the fit, informed by Bayesian information criterion. See Materials and Methods for more details.

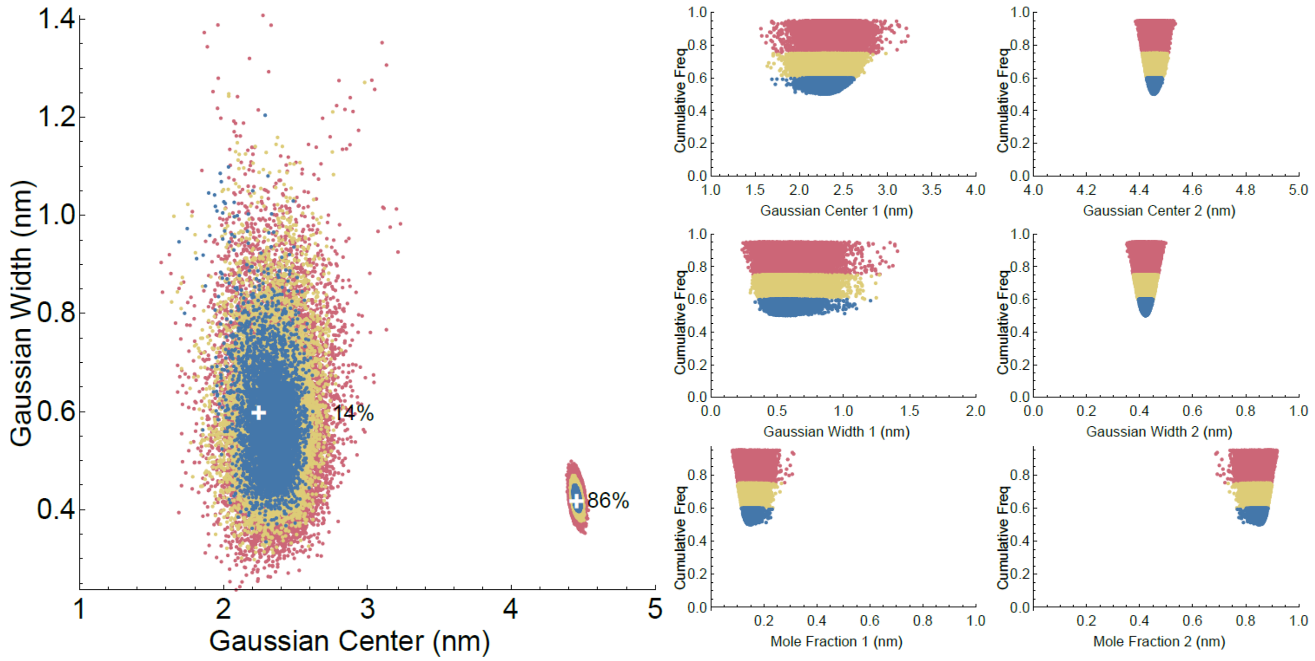

Fig. S5. The Monte Carlo error surface for a 2-Gaussian fit of the double BSL apoCaM sample data, with the centers, widths and mole fractions simultaneously being varied. The 60%, 75% and 95% confidence intervals (blue, yellow, and red, respectively) were determined by a taking a ratio of the residual sum of squares of all fits with the residual sum of square of the best found fit using the cumulative distribution function of the F-ratio distribution with the appropriate degrees of freedom given the amount of data and the number of varied parameters. White crosses denote the location of the best found fit with the listed mole fractions.

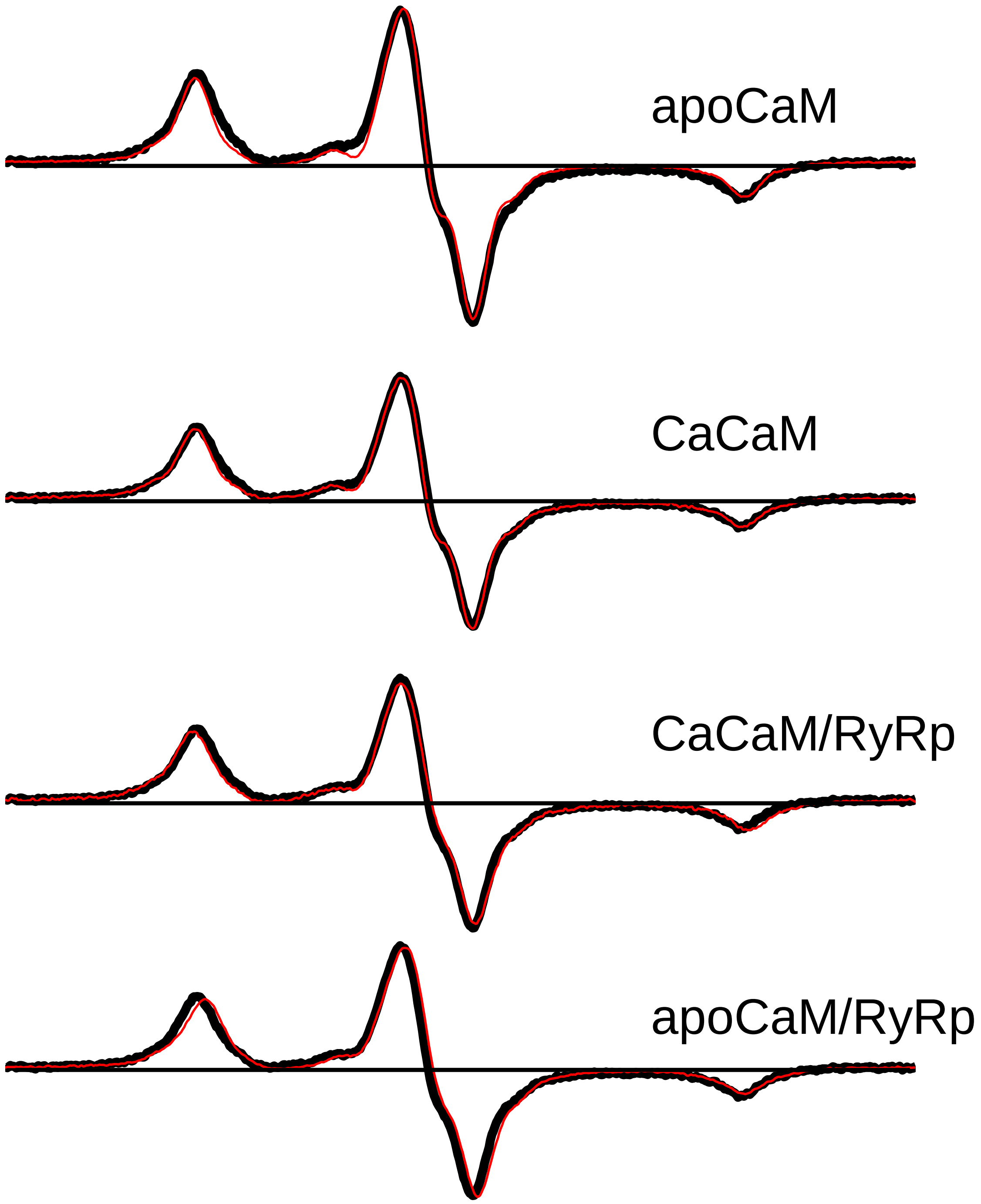

Fig. S6. Dipolar CW EPR of double BSL labeled CaM at positions T34CS38C in the N-lobe and R106CS110C in the C-lobe. Overlay shows double labeled construct (red) with single labeled construct (black), obtained by averaging spectra of the two single labeled samples. The lack of broadening indicates that there is no spin-spin interaction within 2.0 nm in each of the experimental conditions, indicating that all structural states populated by CaM were captured in the DEER measurements. Experiments were performed at 200 K. All spectra are normalized to the spin concentration by dividing by the double integral. Scan width obtained at 200 G (shown as 120 G).

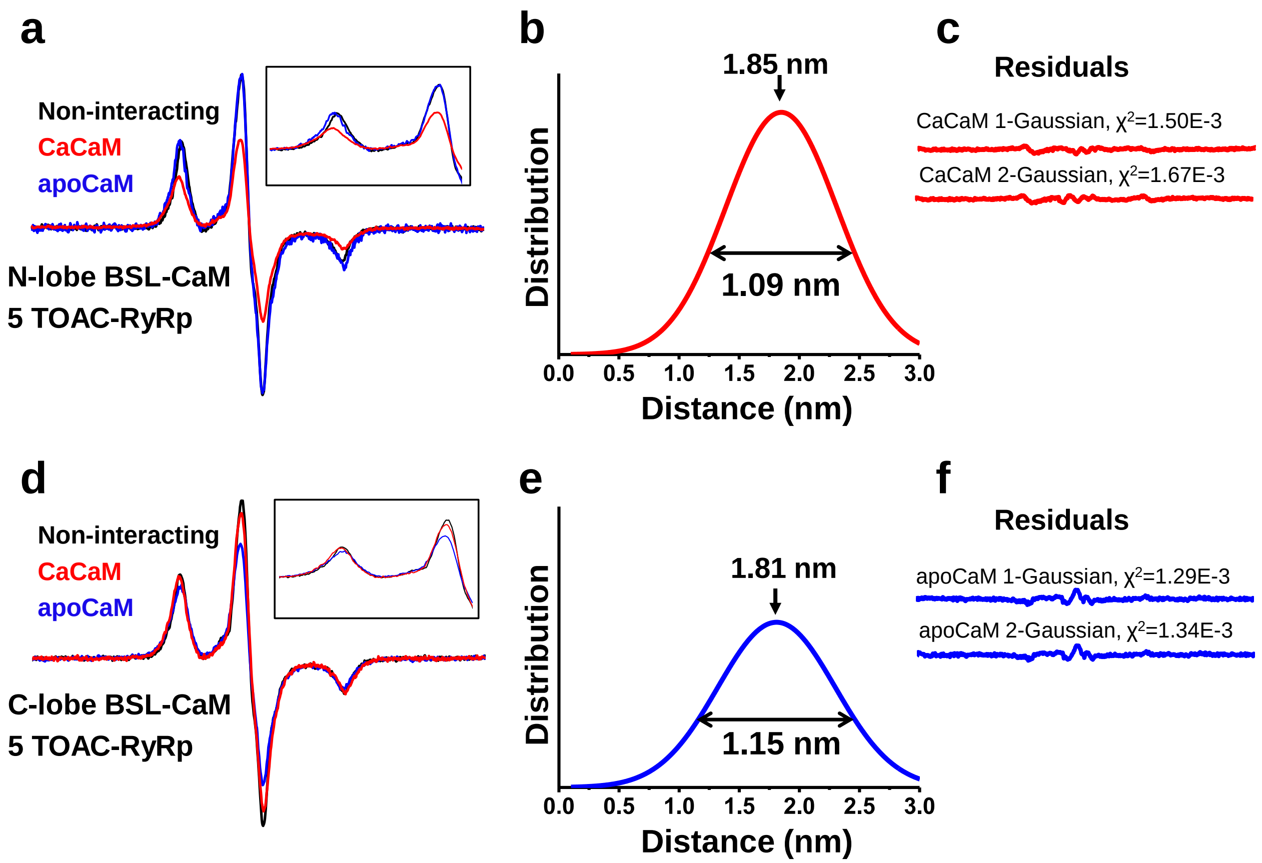

Fig. S7. Analysis of the dipolar CW EPR data of N-lobe (top) or C-lobe (bottom) BSL-CaM/TOAC-RyRp. (**a** and **d**) Representative EPR spectra shown at 200 G scan width. Each spectrum has been normalized to the same number of spins by dividing by the double integral. Inset shows expanded view of a 50-G portion of the low-field. The non-interacting spectrum was obtained by averaging spectra of the two singly-labeled samples, prepared from single-Cys mutants. Only the spectrum that showed broadening was analyzed. (**b** and **e**) Distance distribution from fits to EPR spectra. (**c** and **f**) Residuals show that the data was best fitted to 1-Gaussian distribution in both cases.

Table S1. Interprobe center distances R, FWHM, and percent mole fractions observed in each biochemical state of double labeled BSL-CaM, detected by DEER

Parameter	apoCaM	CaCaM	CaCaM/RyRp	apoCaM/RyRp
**Fraction$_1$ (%)**	15 ± 12	29 ± 11	87 ± 13	17 ± 11
**R_1 (nm)**	2.4 ± 0.8	1.6 ± 0.3	2.2 ± 0.1	2.4 ± 0.7
**FWHM$_1$ (nm)**	1.4 ± 1.4	1.6 ± 0.8	0.7 ± 0.2	1.5 ± 1.2
**Fraction$_2$ (%)**	85 ± 12	28 ± 10	13 ± 13	83 ± 11
**R_2 (nm)**	4.5 ± 0.1	4.1 ± 0.2	3.0 ± 0.8	4.5 ± 0.1
**FWHM$_2$ (nm)**	1.0 ± 0.2	0.8 ± 0.4	1.2 ± 1.2	1.3 ± 0.2
**Fraction$_3$ (%)**	n/a	44 ± 7	n/a	n/a
**R_3 (nm)**	n/a	5.7 ± 0.1	n/a	n/a
**FWHM$_3$ (nm)**	n/a	1.1 ± 0.2	n/a	n/a

R, mean distance; FWHM; full width of the Gaussian distribution at half maximum (*see Materials and Methods* Eqs 1 and 2.); Fraction, percent of mole fraction of CaM occupying a given structural state. Subscript indicates population number. Errors represent 95% confidence interval obtained from an F-test of the Monte Carlo search results (see Fig. S5). Experimental conditions are described in *Materials and Methods.*

Table S2. Interprobe center distances R, FWHM, and percent mole fractions observed in each biochemical state of single labeled N-lobe or C-lobe BSL CaM complexed with 5 TOAC RyRp, detected by DEER

Population Parameters	N-lobe -Ca	N-lobe +Ca	C-lobe -Ca	C-lobe +Ca
**Fraction$_1$ (%)**	75 ± 18	97 ± 8	100 ± 0	89 ± 9
**R_1 (nm)**	2.3 ± 0.6	2.3 ± 0.1	1.5 ± 0.2	2.2 ± 0.1
**FWHM$_1$ (nm)**	2.4 ± 1.3	0.8 ± 0.1	2.0 ± 0.3	0.9 ± 0.1
**Fraction$_2$ (%)**	25 ± 18	3 ± 8	n/a	11 ± 9
**R_2 (nm)**	4.2 ± 0.2	3.3 ± 0.9	n/a	2.5 ± 0.1
**FWHM$_2$ (nm)**	1.1 ± 0.5	0.4 ± 0.9	n/a	0.3 ± 0.2

R, mean distance; FWHM; full width of the Gaussian distribution at half maximum (*see Materials and Methods* Eqs 1 and 2.); Fraction, mole fraction of CaM occupying a given structural state. Subscript indicates population number. Errors represent 95% confidence interval obtained from an F-test of the Monte Carlo search results (see Fig. S5). Experimental conditions are described in *Materials and Methods.*

Table S3. Parameters from CW EPR analysis of BSL-CaM and BSL-CaM/RyRp labeled at positions T34CS38C in the N-lobe or R106CT110C in the C-lobe, as indicated

	N-lobe			C-lobe		
**Sample**	**$2T_{II}'$ (G)**	**τ_R (ns)**	**S**	**$2T_{II}'$ (G)**	**τ_R (ns)**	**S**
CaCaM	65.5 ± 0.15	10.24 ± 0.23	0.79 ± 0.004	65.0 ± 0.12	9.48 ± 0.17	0.78 ± 0.003
apoCaM	64.4 ± 0.17	8.73 ± 0.22	0.76 ± 0.004	64.3 ± 0.19	8.61 ± 0.23	0.76 ± 0.005
CaCaM/RyRp	65.9 ± 0.10	10.90 ± 0.32	0.80 ± 0.002	64.7 ± 0.20	9.08 ± 0.26	0.77 ± 0.005
apoCaM/RyRp	65.5 ± 0.12	10.41 ± 0.19	0.79 ± 0.003	65.5 ± 0.15	10.20 ± 0.24	0.79 ± 0.003

$2T_{II}'$, splitting between the outer extrema, τ_R, rotational correlation time determined empirically from splitting (Eq. 3), S, order parameter determined from splitting (Eq. 4). Each value is mean and standard error from multiple experiments (n = 3).
